# Adopting Clinical Practice Guidelines for Pharmacologic Management of Acute Spinal Cord Injury from a Developed World Context to a Developing Global Region

**DOI:** 10.34172/aim.2022.58

**Published:** 2022-06-01

**Authors:** Seyed Behnam Jazayeri, Seyed Farzad Maroufi, Zahra Ghodsi, Heshmatollah Ghawami, Ahmad Pourrashidi, Abbas Amirjamshidi, Mojtaba Mojtahedzadeh, Jalil Arabkheradmand, Farzin Farahbakhsh, Maryam Shabany, Morteza Faghih-Jouibari, Michael G. Fehlings, Brian K. Kwon, James S. Harrop, Vafa Rahimi-Movaghar

**Affiliations:** ^1^Sina Trauma and Surgery Research Center, Tehran University of Medical Sciences, Tehran, Iran; ^2^Faculty of Medicine, Tehran University of Medical Sciences, Tehran, Iran; ^3^Brain and Spinal Cord Injury Research Center, Neuroscience Institute, Tehran University of Medical Sciences, Tehran, Iran; ^4^Neuropsychology Division, Sina Trauma and Surgery Research Center, Tehran University of Medical Sciences, Tehran, Iran; ^5^Sina Hospital, Tehran University of Medical Sciences, Tehran, Iran; ^6^Director of Fellowship Program in Clinical Pharmacy and Critical Care, Faculty of Pharmacy; ^7^Director, Defence Health Research C, Tehran, Iran; ^8^Sports Medicine Research Center, Neuroscience Institute, Tehran University of Medical Sciences, Tehran, Iran; ^9^Department of Neurosurgery, Shariati Hospital, Tehran University of Medical Sciences, Tehran, Iran; ^10^University of Toronto Spine Program and Toronto Western Hospital, Toronto, Ontario, Canada; ^11^International Collaboration on Repair Discoveries (ICORD), University of British Columbia, Vancouver, BC, Canada; ^12^Department of Orthopaedics, Division of Spine, University of British Columbia, Vancouver, BC, Canada; ^13^Department of Neurological and Orthopedic Surgery, Thomas Jefferson University, Philadelphia, PA, USA; ^14^Universal Scientific Education and Research Network (USERN), Tehran, Iran; ^15^Institute of Biochemistry and Biophysics, University of Tehran, Tehran, Iran; ^16^Visiting Professor, Spine Program, University of Toronto, Toronto, Canada

**Keywords:** Clinical practice guideline, Pharmacologic management, Spinal cord injury

## Abstract

**Background:**

Proper utilization of high-quality clinical practice guidelines (CPGs) eliminates the dependence of patients’ outcomes on the ability and knowledge of "individual" health care providers and reduces unwarranted variation in care. The aim of this study was to adapt/adopt two CPGs for pharmacologic management of acute spinal cord injury (SCI) using guideline adaptation methods.

**Methods:**

This study was conducted based on the ADAPTE process. Following establishment of an organizing committee and choosing the health topics, we appraised the quality of the CPGs using the Appraisal of Clinical Guidelines for Research & Evaluation II (AGREE II). Then, the authors extracted and categorized suggestions according to Population, Intervention, Professions, Outcomes and Health care setting (PIPOH). The decision-making process was based on systemic evaluation of each suggestion, utilizing a combination of AGREE II scores, the quality of supporting evidence for or against each suggestion and the triad of feasibility, acceptance and adoptability for the Iranian health-care context.

**Results:**

Two guidelines were included in the adaptation process. Based on high-quality of these guidelines and the feasibility and adoptability evaluation of the organizing committee, we decided to adopt the suggestion of both guidelines. Overall, seven suggestions were extracted from the source guidelines.

**Conclusion:**

This work provides a framework to apply guidelines for acute SCI to the developing regions of the world. Attempts should be made to implement these suggestions in order to improve the health outcomes of Iranian SCI patients.

## Introduction

 Spinal cord injury (SCI) is a devastating and long-lasting condition that harms the normal sensory, motor and autonomic functions of patients and has a significant worldwide health and social impact.^1^ The annual health-care costs for someone with SCI are estimated to be up to six times higher than individual suffering from other chronic conditions.^2^ The estimated lifetime direct costs for an individual injured at age 25 ranges on average from 1.1 to 3.5 million dollars based on the level and severity of injury.^3^

 Many of the secondary complications and deleterious consequences of SCIs are not only associated with the impact from the injury itself, but by also by challenges in the delivery of appropriate medical and rehabilitation services.^4^ Six essential measures have been proposed by the World Health Organization (WHO) to help people with SCI live longer and healthier and have more social participation: (1) Timely, appropriate pre-hospital management; (2) Acute critical care relevant to the severity and type of injury (including surgical and pharmacologic interventions); (3) Access to continued health care, and health-related education and products; (4) Access to skilled rehabilitation and mental health services; (5) Access to appropriate assistive devices; and 6. Specialized proficiency and knowledge among SCI-care providers.^5^ Most of these measures can improve with access to evidence-based clinical practice guidelines (CPGs) and decision-making support tools for SCI care providers.

 CPGs are “systematically developed statements to aid practitioners and patients in making decisions regarding appropriate health care for specific clinical conditions”.^6^ Indeed, proper utilization of high-quality CPGs eliminates the dependence of patient’s outcomes on the ability and knowledge of “individual” health care providers and reduces unwarranted variation in care. Development of a *de novo* CPG is usually costly and time-consuming, and requires special panels of researchers and experts who systematically evaluate the body of evidence to develop a comprehensive and feasible evidence-based guideline.^7^ Such a process is often outside the budget of developing countries; therefore, a more efficient strategy to centralize care in developing countries can be adapting CPGs generated in developed countries.

 In 2017, a multidisciplinary guideline development group developed five CPGs for the management of acute SCI.^8^ These guidelines provide evidence-based suggestions for SCI management including the focus of this study, pharmacological management of acute SCI. For the purpose of this project, the pharmacologic management of SCI refers to the proper use of methylprednisolone sodium succinate (MPSS) and the type and timing of anticoagulation prophylaxis in patients with SCI. MPSS is a corticosteroid that has useful implications in pathophysiology of SCI because of its anti-inflammatory properties. However, the drug is not Food and Drug Administration (FDA) approved for application in SCI and there are some issues about its safety profile.^9,10^

 Patients with SCI also have higher risks of thromboembolic events such as deep vein thrombosis (DVT) and pulmonary embolism (PE), which are significant sources of morbidity and mortality in SCI patients.^11^ Application of the “recommendations and/or suggestions” in the depicted guidelines provides an opportunity for harmonization and integration of SCI care management, specifically in developing countries such as Iran. Therefore, the objective of this study was to adapt/adopt two high-quality CPGs for pharmacologic management of acute SCI using a systematic and evidence-based approach.

## Materials and Methods

###  Study Design

 We designed and conducted the adaptation process based on the ADAPTE methodology. The ADAPTE process provides systematic instructions for adapting guidelines developed in one health-care context for implementation in a different context.^12^ This process ensures that the final recommendations address the particular health concerns that are relevant, taking into consideration the priorities, legislations, policies, and resources of the targeted setting. ADAPTE was chosen as it represents the most structured and commonly used approach for the adaptation process.^13^ ADAPTE is made of three phases (set-up, adaptation, and finalization), 9 modules, and 24 steps. We also decided to add an extra phase to the ADAPTE process in order to facilitate the dissemination and implementation of guidelines by setting a series of podcasts. Table 1 summarizes the phases and steps of our method for adaptation of the SCI guidelines sourced by the ADAPTE process.

**Table 1:** ADAPTE Process to Guideline Adaptation

**Phases**	**Steps**
Set–up phase	**1.Establish an organizing committee**: The organizing committee was consisted of an executive committee and an expert panel. The executive committee included 7 medical students, a mentor and a project manager. The expert panel consisted of, 9 neurosurgeons (5 Iranian and 4 international), 1 clinical pharmacist, 1 trauma experts, 1 radiologist, 1 rehabilitation nurse and 1 general practitioner.
	**2. Select a topic** We chose two topics: A: A clinical practice guideline for the management of patients with acute spinal cord injury: recommendations on the use of MPSS.^9^ B: A clinical practice guideline for the management of patients with acute spinal cord injury: recommendations on the type and timing of anticoagulant thromboprophylaxis.^10^
	**3. Check whether adaptation is feasible ** Based on internal and external validity checking of guidelines we assumed that the adaptation is feasible.
	**4. Identify skills and resources needed** Our team had the experience of translation, and adaptation of traumatic brain injury guidelines. We used the knowledge and experience of that committee for our topics.
	**5. Complete set–up tasks**
	**6. Write protocol**
Adaptation phase	**7. Determine the health questions** Should MPSS be routinely used in adult (> 14 years) Iranian patients with acute SCI? Should anticoagulant thromboprophylaxis be used in the acute period after SCI?
	**8. Search for guidelines and other potential documents**
	**9.Screen retrieved guidelines**
	**10. Reduce a large number of retrieved guidelines**
	**14. Assess guideline consistency ** The consistency of guidelines was checked by careful evaluation of the process of developing recommendations by the source guideline including: The search strategy, method of data extraction from retrieved sources, method of data summarization and interpretation of the evidence, the level of supporting evidence and the consistency between the interpretation of the evidence and the recommendations.
	**15. Assess acceptability/applicability of the recommendations:** The panel checked the acceptability/applicability of the recommendations based on evaluation of the differences between the organizational and cultural context of the source guidelines with Iranian healthcare setting, including the resources, accessibility of health services, and characteristics of the Iranian population such as their traditional beliefs and value judgments.
	**16. Review assessments to aid in decision–making:** Panel members were presented with all documents that summarized the results of the assessment module
	**17. Select between guidelines and recommendations to create an adapted guideline:** Decision-making and selection occurred around the following five options: 1) REJECT the whole guideline 2) ACCEPT the whole guideline and all of its recommendations 3) ACCEPT the evidence summary of the guideline 4) ACCEPT specific recommendations 5) MODIFY specific recommendations
	**18. Prepare a document that respects the needs of the end users and provides a detailed transparent explanation of the process**
Finalization phase	**19. External review by target users**
	**20. Consult with relevant endorsement bodies** After adaptation we shared our results with our international experts for their feedbacks on the work.
	**21. Consult with developers of source guidelines** We sent the draft of our work to the guideline development group of the original articles for feedback on the work.
	**22. Acknowledge source documents**
	**23. Plan for aftercare of the adapted guideline**
	**24. Produce high quality final guideline**

SCI, spinal cord injury.

###  Set-up Phase (Steps #1-6 ADAPTE)

 At the onset of the study in 2019, we had predetermined the guideline topics (i.e. ADAPTE step #2) and decided to rely on two specific guidelines instead of searching for all the existing literature. This decision was based on three reasons. First, these guidelines had utilized a systematic review to answer a specific clinical-relevant area of controversy in the literature, and hence summarized previous published guidelines, and specified the knowledge gaps about each topic. Second, a systematic and high-quality approach was used to develop suggestions and/or recommendations in these guidelines. Third, the guidelines had undergone an assessment of both internal and external validity prior to publication.^14^

 Establishment of organizing committees (i.e. ADAPTE step #1): We set up an executive committee which contained seven medical students responsible for translation and management of the adaptation process of guidelines and also for podcast set-up. The teams had a mentor (SBJ) whose responsibility was to monitor the team’s function and coordinate the expert panel meetings. They were supported by a project coordinator (ZGh) who helped with communication with the expert panel, and meeting organization. The expert panel consisted of stakeholders affected by the guidelines (i.e. ADAPTE step #4). They had the clinical knowledge and personal experience as well as policy administrative and methodological expertise in the topic areas. The expert panel consisted of 14 people including 9 neurosurgeons (5 Iranian and 4 international), 1 clinical pharmacist, 2 trauma experts, 1 rehabilitation nurse and 1 general practitioner. The expert panel was responsible for adapting the recommendation and/or suggestions of the source guidelines based on communities’ support needs. The outline of the project (steps #5 to 6) was established by the executive committee and confirmed by the committee during an online meeting. All members signed a declaration of conflict of interest form.

###  Adaptation Phase (Steps #7-15 ADAPTE)

 The first step was defining specific health questions addressed by each guideline (step #7). We considered the Population, Intervention, Professions, Outcomes and Healthcare setting (PIPOH) tool to summarize the population, intervention, professions, outcomes and health setting of each guideline. The target population of the guidelines are adult patients (more than 14 years of age) with acute, blunt traumatic SCI who have American Spinal Injury Association (ASIA) grades A to D after resuscitation. Table 2 summarizes the PIPOH tabled by the executive committee in collaboration with the experts.

**Table 2:** PIPOH Evaluation of the Guidelines

**Population**	Adult (> 14 years) patients with acute blunt traumatic SCI ASIA grade A-D
**Intervention**	Use of a 24-hour infusion of high-dose MPSS Use of a 48-hour infusion of high-dose MPSS Use of anticoagulant thromboprophylaxis Use of either Enoxaparin or Dalteparin Use of either fixed, low-dose or adjusted dose UFH Use of either LMWH (tinzaparin and dalteparin) or UFH
**Professionals**	Neurosurgeons Emergency physician General practitioner Nurse Trauma unit staff
**Outcomes**	Change in motor and sensory scores and risk of major complications Reduced risk of DVT and PE without increased risk of bleeding and mortality
**Health care setting**	Tertiary care

SCI, spinal cord injury; DVT, deep vein thrombosis; PE, pulmonary embolism; UFH, unfractionated heparin; LMWH, low-molecular-weight heparin; PIPOH, Population, Intervention, Professions, Outcomes and Health care.

 The next step was to search for guidelines (steps #8-10). As stated, at the onset of the study, we were aware of two quality guidelines for our topics. Therefore, our search was limited to ascertain whether a more recent version of the source guidelines existed.

 The ADAPTE methodology also suggests evaluation of quality (step #11), currency (step #12), content (step #13), consistency (step #14) and acceptability/adaptability of source guidelines (step #15). The quality of the each guideline was independently appraised by four reviewers using the Appraisal of Guidelines for Research and Evaluation (AGREE II) instrument. AGREE II evaluates the methodological rigor and transparency of the development process of guidelines through six domains as follow: scope and purpose, stakeholder involvement, rigor of development, clarity and presentation, applicability and editorial independence. Further evaluation of each suggestion (steps #12-15) was achieved by designing a matrix that contained (1) the AGREE II assessment score of guideline, (2) the body of suggestion and the health question to be answered, (3) the quality of evidence and rational for each suggestion and (4) the strength of each suggestion based on Grading of Recommendations Assessment, Development and Evaluation (GRADE) which was reported in the source guidelines. This matrix was presented to all members of the expert panel and they were asked to value each suggestion based on (1) feasibility (existence of appropriate infrastructure to implement the suggestion; including equipment, technology, and other facilities), (2) relevance (matching the characteristics of the described clinical population with the Iranian population), (3) acceptability (compliance with patients’ preferences in the country, in accordance with the culture and customs of the community and cost-effectiveness of intervention), and (4) overall adoptability. We used a 3-point Likert scale (Agree, Disagree, and Undecided) to measure the experts’ attitudes.

###  Decision-making (steps #16-17) 

 The process of decision-making was based on (1) AGREE II scores (2) suggestion assessments (steps #11-15) and (3) expert panel comments. A consensus method was used to decide the procedure of selecting suggestions for adaption. As suggested by the ADAPTE collaboration, decision-making and selection (step #17) occurred around the following five options: 1. Completely reject the guideline; 2. Completely accept the guideline and its suggestions; 3. Accept the evidence summary; 4. Accept the specific suggestions; and 5. Modify specific suggestions. The final step in the adaptation phase (step #18) was preparation of the adapted guideline.

## Results

**Figure 1:**
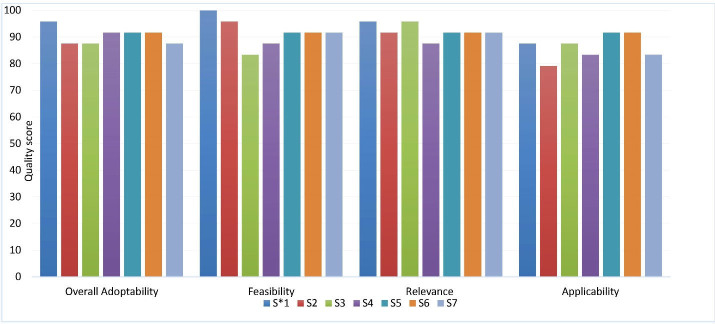
Quality Assessment of the Source Guidelines. Each suggestion was assessed by members of the expert panel in a structured survey. The survey contained questions about feasibility, relevance, applicability and overall adoptability of each suggestion in a 3-point Likert scale format. The table shows the percentage of agreement for each category. S1: Anticoagulant thromboprophylaxis should be offered routinely (if possible, within the first 72h after injury) to reduce the risk of thromboembolic events in the acute period after SCI. S2: Eighter subcutaneous LMWH (Enoxaparin or Dalterparin) can be used to reduce the risk of thromboembolic events in the acute period after traumatic SCI. S3: Fixed-dose UFH should be used to reduce the risk of thromboembolic events in the acute period after traumatic SCI. The use of adjusted-dose UFH should be prohibited due to increased risk of bleeding. S4: Both LMWH and fixed-dose UFH can be used to reduce the risk of thromboembolic events in the acute period after traumatic SCI. S5: MPSS should not be administered to adult patients with acute SCI after 8 hours after injury. S6: A 48-hour infusion of MPSS is not suggested for adult patients with acute SCI after 8 hours after injury. S7: A 24-hour infusion of high-dose MPSS should be administered to adult patients with acute SCI within 8 hours of injury. S* = suggestion.

 Both selected guidelines were developed under the auspices of AOSpine North America, AOSpine International, the American Association of Neurological Surgeons and the Congress of Neurological Surgeons (AANS/CNS). The first guideline contained suggestions on the optimal type and timing of anticoagulant prophylaxis in acute SCI (anticoagulant guideline) and the second guideline addressed issues on MPSS use in the acute setting of SCI (MPSS guideline). The overall scores for the rigor of development domain from AGREE II assessment were 96.2% and 94% for the Anticoagulant and MPSS guidelines, respectively. In the current study, more than 80% of the panel rated high for relevancy, acceptability/applicability and feasibility of the recommendations. Based on the experts’ comments, the rationale for relevancy ratings were based on evaluation of the current evidence, its consistency with prior knowledge and the experience of the panelists. The ratings for the applicability of the recommendations were based on evaluation of the differences between the organizational and cultural context of the source guidelines with Iranian healthcare setting, including the resources, accessibility of health services, and characteristics of the Iranian population such as their traditional beliefs and value judgments. Overall, the committee decided to adopt the suggestions of both guidelines because of their high score (> 85%) for overall adoptability (Figure 1). However, the expert panel admitted that there are some challenges to implementation and dissemination of these suggestions by target users.

 Finally, seven suggestions were extracted from the source guidelines. The level of evidence was low for four suggestions and moderate for two suggestions. One suggestion was purely based on experts’ opinion. Table 3 summarizes the suggestions and their level of evidence.

**Table 3:** List of Final Recommendations and Their Level of Evidence

**Question**	**P**	**I**	**C**	**O**	**Level of Evidence **	**Recommendation**
Should anticoagulant thromboprophylaxis be offered to reduce the risk of thromboembolic events in the acute period after SCI in Iran?	Patients with acute SCI	Use of anticoagulant thromboprophylaxis	Prophylaxis with No prophylaxis or placebo	Reduced risk of DVT and PE without increased risk of bleeding and mortality	Low	The panel suggests that anticoagulant thromboprophylaxis be offered routinely (if possible, within the first 72 h after injury) to reduce the risk of thromboembolic events in the acute period after SCI.
What anticoagulant thromboprophylaxis should be employed to reduce the risk of thromboembolic events in the acute period after traumatic SCI in Iran? A: Should enoxaparin versus dalteparin be used to reduce the risk of thromboembolic events in the acute period after traumatic SCI?	Patients with acute SCI	Use of either Enoxaparin or Dalteparin	Enoxaparin with Dalteparin	Reduced risk of DVT and PE without increased risk of bleeding and mortality	Low	The panel suggests that either subcutaneous LMWH can be used to reduce the risk of thromboembolic events in the acute period after traumatic SCI.
Should fixed, low-dose versus adjusted-dose UFH be used to reduce the risk of thromboembolic events in the acute period after traumatic SCI?	Patients with acute SCI	Use of either fixed, low-dose or adjusted dose UFH	fixed, low-dose with adjusted dose UFH	Reduced risk of DVT and PE without increased risk of bleeding and mortality	Low	The panel suggests that fixed-dose UFH be used to reduce the risk of thromboembolic events in the acute period after traumatic SCI. The use of adjusted-dose UFH should be prohibited due to increased risk of bleeding.
Should LMWH versus UFH be used to reduce the risk of thromboembolic events in the acute period after traumatic SCI?	Patients with acute SCI	Use of either LMWH (Tinzaparin and Dalteparin) or UFH	LMWH with UFH	Reduced risk of DVT and PE without increased risk of bleeding and mortality	Low	The panel suggests that both LMWH and fixed-dose UFH can be used to reduce the risk of thromboembolic events in the acute period after traumatic SCI
Should a 24-hour infusion of high-dose MPSS be administered to adult patients with acute SCI after 8 hours after injury?	Patients with acute SCI after 8 hours of injury	Use of a 24-hour infusion of high-dose MPSS	MPSS with no treatment	Change in motor and sensory scores and risk of major complications	Moderate	The panel suggests not administering MPSS to adult patients with acute SCI after 8 hours after injury
Should a 48-hour infusion of high-dose MPSS be administered to adult patients with acute SCI?	Patients with acute SCI	Use of a 48-hour infusion of high-dose MPSS	24-hour vs 48-hour MPSS infusion	Change in motor and sensory scores and risk of major complications	No Study	The panel suggests not administering a 48-hour infusion of MPSS to adult patients with acute SCI after 8 hours after injury.
Should a 24-hour infusion of high-dose MPSS be administered to adult patients with acute SCI within 8 hours of injury?	Patients with acute SCI within 8 hours of injury	Use of a 24-hour infusion of high-dose MPSS	MPSS with no treatment	Change in motor and sensory scores and risk of major complications	Moderate	The panel suggests a 24-hour infusion of high-dose MPSS to be administered to adult patients with acute SCI within 8 hours of injury.

SCI, spinal cord injury; DVT, deep vein thrombosis; PE, pulmonary embolism; UFH, unfractionated heparin; LMWH, low-molecular-weight heparin; MPSS, methylprednisolone sodium succinate.

## Discussion

 SCI management has changed drastically during the past decade due to the advancement in understanding the mechanisms and pathophysiology of SCI. The appropriate use of corticosteroids and the type and timing of anticoagulation prophylaxis are two major topics in the literature that have gained significance in most recent guidelines.^9,10,14^ Developing guidelines for pharmacologic management in patients with acute SCI has been attempted repeatedly, including the 2002 and 2013 AANS/CNS- CPGs.^15,16^ In the guideline published in 2002, the authors state that there is not sufficient evidence to support treatment standards and guidelines for the use of MPSS in the context of SCI. In 2017, re-examination of existing evidence clarified the controversy surrounding the use of MPSS in patients with acute SCI. The authors suggested “*24-hour infusion of high-dose MPSS merely to adult patients who present within 8 hours of acute SCI*”.^9^ Furthermore regarding anticoagulant therapy, the following suggestions were developed in the 2017 guideline: 1. “*the authors suggested that anticoagulant thromboprophylaxis be offered routinely to reduce the risk of thromboembolic events in the acute period after SCI*”; 2. They suggested that “*anticoagulant thromboprophylaxis, consisting of either subcutaneous low-molecular-weight heparin (LMWH) or fixed, low-dose unfractionated heparin (UFH), should be offered to reduce the risk of thromboembolic events in the acute period after SCI*”; 3. “*Given the potential for increased bleeding events with the use of adjusted-dose UFH, the authors suggest against this option*” and 4. “*The authors suggested commencing anticoagulant thromboprophylaxis within the first 72 hours after injury, if possible, in order to minimize the risk of venous thromboembolic complications during the period of acute hospitalization*”.^10^

 In this study, we decided to adopt the suggestions of the two abovementioned guidelines. This decision was made based on systemic evaluation of each suggestion utilizing AGREE II scores, the quality of supporting evidence for or against each suggestion and the triad of feasibility, acceptance and adoptability in the Iranian health-care context. Although the level of evidence for the majority of suggestions was low, the expert panel reached the consensus that implementing these suggestions is an efficient way with the potential to promote the health of SCI patients. However, we are aware that there are many challenges that affect the implementation of CPGs. Cultural relevance, availability, cost, equity, access to treatment, and many other factors must be considered by local policymakers, physicians, and/or patients. It should also be noted that CPGs may be subject to modification over time in order to provide best available suggestions based on the most up-to-date research. The Guideline Development Group (GDG) of the source guidelines have stated that they have a plan for updating the guidelines every 3-5 years or even earlier if there are changes in *1. The evidence related to harms and benefits; 2. Outcomes that would be considered important for decision making; 3. Ranking of current critical and important outcomes; and 4. Available interventions and resources*. At the time of finalizing the manuscript, we checked the guidelines’ publisher website for possibility of an update guideline and also asked the GDG by email about the existence of a newer version of guidelines and ensured that there is no update version for the guidelines.

 While adapting the source guidelines and based on the experience of the expert panel, we recognized the importance of the uptake of these suggestions by clinical practitioners. For this purpose, we aim to set up podcasts. The essence of podcasting is to create content (audio or video podcasts) for an audience that wants to listen whenever and wherever.^17^ In fact, learners perceive podcasts to be a more beneficial resource over traditional books and journals.^18^ By recording key topics as downloadable content, we can create podcasts that will easily and conveniently reach our target audience. We have negotiated our aims of this study in the global spine congress 2019 with the authors of the original guidelines and we have sought the support of the international guideline development group for adaptation and adoption of the guidelines. We also have a plan to initiate uploading international podcasts for the AOSpine SCI knowledge forum with the support of AOSpine SCI members.

 The main finding of our study was that the suggestions developed by the auspices of AOSpine North America, AOSpine International, and the AANS/CNS guidelines are adoptable in a developing country (Iranian healthcare setting). Adaptation of CPGs from developed regions of the world to developing economies is challenging. This article sets out a framework to apply high quality guidelines for the pharmacologic management of acute SCI to a developing region of the world (Iran). This work has considerable significance for other developing regions of the world who seek to advance the care pathways for individuals with an acute SCI.
